# On Easiest Functions for Mutation Operators in Bio-Inspired Optimisation

**DOI:** 10.1007/s00453-016-0201-4

**Published:** 2016-08-18

**Authors:** Dogan Corus, Jun He, Thomas Jansen, Pietro S. Oliveto, Dirk Sudholt, Christine Zarges

**Affiliations:** 1 Department of Computer Science, Aberystwyth University, Aberystwyth, SY23 3DB UK; 2 Department of Computer Science, University of Sheffield, Sheffield, S1 4DP UK

**Keywords:** Running time analysis, Theory, Hybridisation, Evolutionary algorithms, Artificial immune systems

## Abstract

Understanding which function classes are easy and which are hard for a given algorithm is a fundamental question for the analysis and design of bio-inspired search heuristics. A natural starting point is to consider the easiest and hardest functions for an algorithm. For the (1+1) EA using standard bit mutation (SBM) it is well known that OneMax is an easiest function with unique optimum while Trap is a hardest. In this paper we extend the analysis of easiest function classes to the contiguous somatic hypermutation (CHM) operator used in artificial immune systems. We define a function MinBlocks and prove that it is an easiest function for the (1+1) EA using CHM, presenting both a runtime and a fixed budget analysis. Since MinBlocks is, up to a factor of 2, a hardest function for standard bit mutations, we consider the effects of combining both operators into a hybrid algorithm. We rigorously prove that by combining the advantages of *k* operators, several hybrid algorithmic schemes have optimal asymptotic performance on the easiest functions for each individual operator. In particular, the hybrid algorithms using CHM and SBM have optimal asymptotic performance on both OneMax and MinBlocks. We then investigate easiest functions for hybrid schemes and show that an easiest function for a hybrid algorithm is not just a trivial weighted combination of the respective easiest functions for each operator.

## Introduction

Over the past years many bio-inspired search heuristics such as evolutionary algorithms, swarm intelligence algorithms, or artificial immune systems, have been developed and successfully applied to various optimisation problems. These heuristics have different strengths and weaknesses in coping with different fitness landscapes. Determining which heuristic is the best choice for a given problem is a fundamental and important question.

We use rigorous theoretical analyses to contribute to a better understanding of bio-inspired algorithms. A natural step is to investigate which functions are easy and which are hard for a given algorithm as this yields fundamental insights into their working principles, particularly with respect to their strengths and weaknesses.

Knowing how well an algorithm performs on its easiest and hardest functions, with regards to the expected optimisation time, provides general insights, as the expected optimisation time of *every* function will be no less than that of an easiest function and no more than that of a hardest function. With respect to applications, such research may also provide guidelines for the design of benchmarks for experimental studies [10].

For a simple elitist (1+1) evolutionary algorithm (EA) using only standard bit mutations (SBM) Doerr, Johannsen, and Winzen showed that OneMax, a function simply counting the number of ones in a bit string, is the easiest problem among all functions with a single global optimum [8]. This statement generalises to the class of all evolutionary algorithms that only use standard bit mutation for variation [33] as well as higher mutation rates and stochastic dominance [37]. On the other hand, He et al. [10] showed that the highly deceptive function Trap is the hardest function for the (1+1) EA.

In this work we consider typical mutation operators in artificial immune systems where no such results are available. Mutation operators in artificial immune systems [6] usually come with much larger mutation rates than mutation operators in evolutionary algorithms. One such example is the somatic contiguous hypermutation (CHM) operator from the B-cell algorithm (BCA) [23], where large contiguous blocks of a bit string are flipped simultaneously. Previous theoretical work on comparing SBM and CHM has contributed to the understanding of their benefits and drawbacks in the context of its runtime on different problems [14, 17, 18], the expected solution quality after a pre-defined number of steps [21], and in dynamic environments [22]. It is easy to see that hardest functions for CHM with respect to expected optimisation times are those where the algorithm gets trapped with positive probability such that the optimisation time is not finite, e. g., if there exists a second best search point for which all direct mutations to the optimum involve non-contiguous bits [17]. However, it is an open problem and the next step forward to determine what kind of functions are easiest for this type of mutation operator.

The BCA uses standard bit mutations alongside CHM and thus can be considered as a hybrid algorithm combining two mutation operators. More specifically, it uses a population of search points and creates $\lambda$ clones for each of them. It then applies standard bit mutation to a randomly selected clone for each parent search point and subsequently applies CHM to all clones. The interplay between these two operators has been investigated for the vertex cover problem [14].

Hybridisations such as hyper-heuristics [30] and memetic algorithms [27] have become very popular over recent years. But despite their practical success their theoretical analysis is still in its infancy, noteworthy examples being the work in [1, 24, 32, 34]. However, nothing is known about easiest or hardest functions for such algorithms.

The goals of this paper are twofold. We first want to understand what functions are easy for CHM and investigate the performance of CHM and SBM on these functions. Afterwards we consider hybridisations of SBM and CHM and easiest functions for such algorithms. We first use the method by He et al. [10], explained in Sect. 3, to derive an easiest function for CHM which we call MinBlocks. In Sect. 4 we present an analysis of the optimisation time as well as a fixed budget analysis for CHM on MinBlocks. We then show that SBM alone is not able to optimise the constructed function and that a hybridisation of SBM and CHM can have significant advantages (Sect. 5.1). Finally, we investigate properties of easiest functions for such hybrid algorithms (Sect. 5.3).

This journal paper extends a preliminary conference paper [4] in several ways. Firstly, in Sect. 3 it is discussed how the framework for determining easiest and hardest functions is not restricted to (1+1)-style algorithms, but is general enough to apply for a much larger class of algorithms including (1+$\lambda$) EAs [13] and the recently popular (1+($\lambda ,\lambda$)) GA using mutation and crossover [7]. Secondly, the analysis of the advantages of hybridisation in Sect. 5.1 has been considerably extended. Rather than just giving an example of a hybrid algorithm using one out of two operators at each step with constant probability, the analysis has been generalised to allow different hybridisation schemes and an arbitrary number *k* of operators. Finally, in Sect. 5.2 an experimental analysis is presented to shed light on the performance of the algorithms for fitness functions that depend both on the number of ones (i.e., OneMax) and the number of blocks (MinBlocks) in the bit string.

## Preliminaries

We are interested in all strictly elitist (1+1) algorithms with time independent variation operators which we formally define in Algorithm 1 for maximisation problems. We refer to any algorithm belonging to this scheme as a (1+1) $A$. This generalised algorithmic scheme keeps a single solution *x* as a population and creates a single offspring at every generation. The new offspring is accepted if it is strictly fitter than the parent. The algorithm outputs the best found solution once a termination condition is satisfied. Since in this paper we are interested in the expected number of steps required by the algorithm to find an optimal solution, we will assume that the algorithm runs forever and we will call *runtime* the number of fitness function evaluations performed before the first point in time when the optimum is found. 
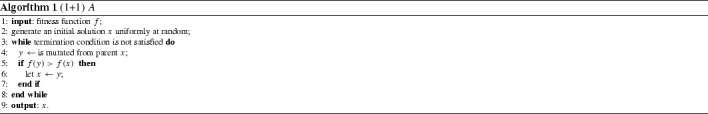


Different algorithms are obtained from the (1+1) $A$ scheme by using different variation operators in line 1 of Algorithm 1. Apart from the explicit requirement of time independence, the variation operators are implicitly restricted to the domain of unary variation operators since the population size is one. In this paper we will consider two different mutation operators that accept a bit string $x\in \{0,1\}^n$ as input. The first is the most common unary variation operator, called standard bit mutation (SBM). This variation operator flips each bit independently with probability 1 / *n*. SBM is formally defined in Algorithm 2. 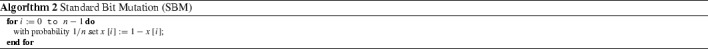


We will refer to the (1+1) $A$ that uses SBM as the (1+1) EA, the most widely studied evolutionary algorithm.

The other variation operator of interest is the contiguous hypermutation operator (CHM), which mutates a bit string by picking a bit position and flipping a random number of bits that follow it (in a wrapping around fashion) each with probability *r*. As done in previous work (see, e. g., [17]), we only consider the extreme case here and set $r=1$. CHM is formally defined in Algorithm 3.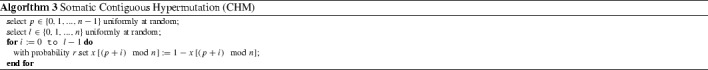 We will refer to the (1+1) $A$ that uses a CHM operator as (1+1) CHM.

Note that Algorithm 1 uses strict selection, i. e. only strict improvements are accepted. For most of our theoretical results we also consider a variant of Algorithm 1 with non-strict selection, where the acceptance condition “$f(y) > f(x)$” is replaced by “$f(y) \ge f(x)$”.

## Easiest and Hardest Functions

Our work is based on previous work by He et al. [10]. We consider the problem of maximising a class of fitness functions with the same optima. An instance of the problem is to maximise a fitness function *f*:

1 $$\begin{aligned} \mathop {\arg \max }\limits _{x \in S} f(x), \end{aligned}$$

where *S* is a finite set.

Let *T*(*A*, *f*, *x*) denote the expected number of function evaluations for the (1+1) $A$ to find an optimal solution for the first time when starting at *x* (*expected hitting time*). In the following we only consider algorithms and functions that lead to a finite expected hitting time.

### Definition 1

[10, Definition 1] Given a (1+1) $A$ for maximising a class of fitness functions with the same optima (denoted by $\mathcal {F}$), a function *f* in the class is said to be an *easiest* function for the (1+1) $A$ if $T(A,f,x) \le T(A, g,x)$ for every $g \in \mathcal {F}$ and every $x \in S$. A function *f* in the class is said to be a *hardest* function for the (1+1) $A$ if $T(A,f,x) \ge T(A, g,x)$ for every $g \in \mathcal {F}$ and every $x \in S$.

The above definition of easiest and hardest functions is based on a point-by-point comparison of the runtime of the EA on two fitness functions. The criteria stated in the following Lemmas 4 and 5 for determining whether a fitness function is an easiest or hardest function for a (1+1) $A$ were originally given in [10]. The main proof idea in [10] is to apply additive drift theorems [11], taking the expected hitting time as drift function (distance to the target state). We state these drift theorems as Theorem 2, referring to the presentation of Lehre and Witt [25], who provided a self-contained proof.

### Theorem 2

(Additive Drift [11, 25]) Let $(X_t)_{t \ge 0}$ be a stochastic process over some bounded state space $S \subseteq \mathbb {R}_0^+$, and let *T* be the first hitting time of state 0. Assume that ${\text {E}\left( T \mid X_0\right) } < \infty$. Then:

(i) If $${\text {E}\left( X_t - X_{t+1} \mid X_0, \dots , X_t; X_t > 0\right) } \ge \delta _u$$ then $${\text {E}\left( T \mid X_0\right) } \le X_0/\delta _u$$.
(ii) If ${\text {E}\left( X_t - X_{t+1} \mid X_0, \dots , X_t\right) } \le \delta _\ell$ then ${\text {E}\left( T \mid X_0\right) } \ge X_0/\delta _\ell$.

Before presenting those criteria, we state and prove the following helper lemma. It is stated as Lemma 3 in [10] without proof.

### Lemma 3

If the expected time *T*(*A*, *f*, *x*) is used as the drift function, then the expected drift^1^ is $\varDelta _f(x)=1$ for all non-optimal search points *x*.

### Proof

Let *P*(*x*, *y*) denote the probability that the search point *y* is adopted as the current search point at the end of an iteration of the (1+1) $A$ with current search point *x*. Since $\sum \limits _{y} P(x,y)=1$,

$$\begin{aligned} T(A,f,x) =\sum \limits _{y} P(x,y)T(A,f,x). \end{aligned}$$

For all non-optimal search points *x*, the (1+1) $A$ will spend one iteration and then continue from the search point reached during this transition, hence

$$\begin{aligned} T(A,f,x) = 1+ \sum \limits _{y} P(x,y)T(A,f,y). \end{aligned}$$

Together, we have

$$\begin{aligned} \sum \limits _{y} P(x,y)T(A,f,x) -\sum \limits _{y} P(x,y)T(A,f,y)&=1\\ \Leftrightarrow \sum \limits _{y} P(x,y)\big (T(A,f,x)-T(A,f,y)\big )&=1\\ \Leftrightarrow \sum \limits _{y} P(x,y)\big (d(x)-d(y)\big )&=1. \end{aligned}$$

That is $\varDelta _f(x) =1$ for all non-optimal search points *x*. $\square$

First, we have the following criterion of determining whether a fitness function is an easiest function for a (1+1) $A$. The two lemmas below extend Theorem 1 and Theorem 2 in [10], respectively, as they are applicable to both strict elitist selection (that is, the parent *x* is replaced by the child *y* if $f(y) > f(x)$) and non-strict elitist selection (that is, the parent *x* is replaced by the child *y* if $f(y) \ge f(x)$). The framework in [10] was restricted to strict selection.

### Lemma 4

Given a (1+1) $A$ with elitist selection (either strict or non-strict) and a class of fitness functions with the same optima, if the following *monotonically decreasing condition* holds,

- for any two points *x* and *y*, if $T(A,f,x) < T(A,f,y)$, then $f(x) > f(y)$,

then *f* is an easiest function in this class.

### Proof

Let *g*(*x*) be any fitness function with the same optima as *f*(*x*). Choose the runtime *T*(*A*, *f*, *x*) as the drift function: $d(x)= T(A,f,x)$.

When maximising *f*(*x*), according to Lemma 3, for any non-optimal point *x* the drift $\varDelta _{f}(x) =1.$ In the following we prove that the drift $\varDelta _{g}(x) \le \varDelta _{f}(x)=1$.

Let $P_f(x, y)$ and $P_g(x,y)$ denote the probability of mutating *x* into *y* (which is independent of the function *f* or *g*) and then *y* being accepted (which is dependent on *f* or *g*) when optimising *f* and *g*, respectively. We separately consider the negative drift $\varDelta _f^-(x) = \sum _{y: d(x) < d(y)} P_f(x, y)(d(x)-d(y))$ and the positive drift $\varDelta _f^+(x) = \sum _{y: d(x) > d(y)} P_f(x, y)(d(x)-d(y))$ and note that $\varDelta _f(x) = \varDelta _f^+(x)+\varDelta _f^-(x)$ as transitions with $d(x)=d(y)$ do not contribute to $\varDelta _f$. The same notation is used for $\varDelta _g$.

Negative drift: let *x* and *y* be two points such that $d(x) < d(y)$. According to the monotonically decreasing condition, $f(x) > f(y)$. For *f*(*x*), since $f(x) > f(y)$, *y* is never accepted and then $\varDelta ^-_{f} (x)= 0.$ But for *g*(*x*), the negative drift is not positive. Thus we have
  2 $$\begin{aligned} \varDelta ^-_{g} (x) \le 0=\varDelta ^-_{f} (x). \end{aligned}$$
Positive drift: let *x* and *y* be two points such that $d(x) > d(y)$. If *y* is an optimum, then naturally $f(x) < f(y)$. If *y* is not an optimum, then according to the monotonically decreasing condition, $f(x) < f(y)$.
  Let $P^{[m]}(x,y)$ denote the probability of mutating *x* into *y* (which is independent of *f* or *g*).
  For *f*(*x*), since $f(x)<f(y)$, *y* is always accepted and then $P_{f}(x,y)=P^{[m]}(x,y).$ But for *g*(*x*), since *g*(*x*) might be larger, smaller than or equal to *g*(*y*), $P_{g}(x,y)\le P^{[m]}(x,y)$. Hence
  $$\begin{aligned} \varDelta ^+_{g}(x) =\;&\sum _{y: d(x)> d (y)} P_{g}( x, y) ( d (x) -d(y)) \\ \le \;&\sum _{y:d (x) >d (y)} P_{f}( x, y) ( d (x) -d(y))=\varDelta ^+_{f}(x). \end{aligned}$$

Considering both negative drift and positive drift, we get ${\varDelta _{g} (x) \le \varDelta _{f}(x)=1}$. Since $\varDelta _{g} (x) \le 1$ for all *x*, according to Theorem 2, the expected runtime is $T(A,g,x) \ge d(x)=T(A,f,x)$ and the theorem statement is derived. $\square$

In a similar way, we have the following criterion of determining whether a fitness function is a hardest function for a (1+1) $A$, assuming that all expected optimisation times are finite^2^. The monotonically decreasing condition in the above lemma is replaced by the monotonically increasing condition.

### Lemma 5

Given a (1+1) $A$ with elitist selection (either strict or non-strict) and a class of fitness functions with the same optima, if the following *monotonically increasing condition* holds,

- for any two non-optimal points *x* and *y*, if $T(A,f,x)< T(A,f,y)$, then $f(x) < f(y)$,

then *f* is a hardest function in this class.

### Proof

Let *g*(*x*) be any fitness function with the same optima as *f*(*x*). Choose the runtime *T*(*A*, *f*, *x*) as the drift function: $d(x)= T(A,f,x)$.

For *f*(*x*), according to Lemma 3, for any non-optimal point *x* the drift $\varDelta _{f}(x) =1.$ For *g*(*x*), we prove that the drift $\varDelta _{g}(x) \ge \varDelta _{f}(x)=1$, using the notation for positive and negative drift from the proof of Lemma 4.

Positive drift: let *x* and *y* be two points such that $d(x) > d(y)$. Let $P^{[m]}(x,y)$ denote the probability of mutating *x* into *y* (which is independent of *f* or *g*). For *f*(*x*), if *y* is an optimum point, then the probability that *x* is mutated into *y* and is accepted, $P_{f}(x,y)$, is equal to $P^{[m]}(x,y)$. Similarly for *g*(*x*), $P_{g}(x,y)=P^{[m]}(x,y)$ if *y* is an optimum point. If *y* is not an optimum point, according to the monotonically increasing condition, $f(x) > f(y)$. For *f*(*x*), since $f(x) > f(y)$, the probability $P_{f}(x,y)=0$. But for *g*(*x*), since *g*(*x*) might be larger, smaller than or equal to *g*(*y*), $P_{g}(x,y)\ge 0$. Thus we have $P_{g}(x,y)\ge P_{f}(x,y)$ for all (*x*, *y*) such that $d(x)>d(y)$. Therefore, the positive drifts for *g* and *f* satisfy
  $$\begin{aligned} \varDelta ^+_{g}(x) =\;&\sum _{y: d(x)> d (y)} P_{g}( x, y) ( d (x) -d(y)) \\ \ge \;&\sum _{y:d (x) >d (y)} P_{f}( x, y) ( d (x) -d(y))=\varDelta ^+_{f}(x). \end{aligned}$$
Negative drift: let *x* and *y* be two points such that $d(x) < d(y)$. Then according to the monotonically increasing condition, $f(x) < f(y)$.
  For *f*(*x*), since $f(x)<f(y)$, *y* is always accepted and then $P_{f}(x,y)=P^{[m]}(x,y).$ But for *g*(*x*), since *g*(*x*) might be larger, smaller than or equal to *g*(*y*), $P_{g}(x,y)\le P^{[m]}(x,y)$. Hence
  $$\begin{aligned} \varDelta ^-_{g}(x) =\;&\sum _{y: d(x)< d (y)} P_{g}( x, y) ( d (x) -d(y)) \\ \ge \;&\sum _{y:d (x) <d (y)} P_{f}( x, y) ( d (x) -d(y))=\varDelta ^-_{f}(x). \end{aligned}$$

Considering both negative drift and positive drift, we get ${\varDelta _{g} (x) \ge \varDelta _{f}(x)=1}$. Since $\varDelta _{g} (x) \ge 1$ for all *x*, according to Theorem 2, the expected runtime is $T(A,g,x) \le d(x)=T(A,f,x)$ and the theorem statement is derived. $\square$

The framework due to He et al. [10] is restricted to search heuristics that base their search on a single point in the search space and it is not obvious how to expand this to population-based algorithms. However, it is worth mentioning that the framework is not restricted to (1+1)-style algorithms. It can also be applied to algorithms that employ elitist selection but have a more complicated way of deciding on the next search point to use as next parameter. Such algorithms can be described as (1+1)-style algorithms with a much more complex mutation operator or, perhaps more adequately, by exactly the same kind of Markov chain as that of a (1+1)-style algorithm, i. e., by a Markov chain of the same size $2^n \times 2^n$. Algorithms where this is true include the (1+$\lambda$) EA (see, e.g., [13]) that creates $\lambda$ offspring by means of mutation, independently and identically distributed, and a best one replaces the current population if its fitness is not worse. In this sense the framework can deal with population-based heuristics (given that the population is restricted to a larger offspring population, not a larger parent population). It can also be applied beyond mutation-based algorithms to genetic algorithms with crossover if the restriction of a population of size only 1 is met. An example for such an algorithm is the so-called (1+($\lambda$, $\lambda$)) GA [7], the first realistic evolutionary algorithm to provably beat the ${\varOmega \left( n\log n\right) }$ lower bound on $\text {OneMax}$. We see that the framework is much more general and useful than it may appear at first sight.

We have already mentioned that we consider functions where an algorithm does not have finite expected optimisation time to be harder than those with finite expected optimisation times. It is well known that CHM (Algorithm 3) can be trapped in local optima when the parameter *r* is set to $r=1$ [2] (see [17, p. 521] for a concrete example demonstrating this effect). Setting $r=1$, however, reveals properties of the hypermutation operator in the clearest way and this is the reason we stick to this choice (compare [17]). This implies that analysing hardest functions for (1+1) CHM does not make much sense because it is easy to find functions where there is a positive probability that the algorithm gets stuck in a local optimum so that, consequently, the expected optimisation time is not finite. One could consider different measures of hardness for this situation, e. g., considering the conditional expected optimisation time given that a global optimum is found or, alternatively, considering the probability not to find a global optimum. This, however, is beyond the scope of this article.

## Contiguous Hypermutations on an Easiest Function with a Unique Global Optimum

We are now ready to derive an easiest function with a unique global optimum for contiguous hypermutations and analyse the performance of the (1+1) CHM on this function.

### Notation and Definition

We use $x = x[0] x[1] \cdots x[n-1] \in \{0, 1\}^n$ as notation for bit strings of length *n*. For $a, b \in \{0, 1, \dots , n-1\}$ we denote by $x[a \dots b]$ the concatenation of *x*[*a*], $x[(a+1) \bmod n]$, $x[(a+2) \bmod n]$, ..., $x[(a+i) \bmod n]$ where *i* is the smallest number from $\{0, 1, \dots , n-1\}$ with $(a+i) \bmod n = b$. We denote by $\left| x[a \dots b]\right|$ the number of bits in $x[a \dots b]$, i. e., its length. We say that $x \in \{0, 1\}^n$ contains a 1-block from *a* to *b* if $x[a \dots b] = 1^{\left| x[a \dots b]\right| }$ and $x[(a-1)\bmod n] = x[(b+1)\bmod n] = 0$ hold. Analogously we may speak of a 0-block from *a* to *b*. Note that bit strings need to contain at least one 0-bit and at least one 1-bit to contain a 0-block or a 1-block. It is easy to see that each $x \in \{0, 1\}^n \setminus \{0^n, 1^n\}$ contains an equal number of 0-blocks and 1-blocks.

#### Definition 6

We define an easiest function with unique global optimum for contiguous hypermutations by defining a partition $L_0 \mathrel {\dot{\cup }} L_1\mathrel {\dot{\cup }} L_2 \mathrel {\dot{\cup }} \cdots \mathrel {\dot{\cup }} L_l = \{0, 1\}^n$ and assigning fitness values accordingly. We call the function $\textsc {MinBlocks}$ and define $\textsc {MinBlocks} (x) = l - i$ for $x \in L_i$. We define $l = \left\lfloor n/2\right\rfloor +1$ and level sets $L_0 = \{ 1^n \}$, $L_1 = \{ 0^n \}$ and $L_i = \large \{ x \in \{0, 1\}^n \mid x$ contains $i-1$ different 1-blocks$\large \}$ for each $i \in \{2, 3, \dots , l\}$.

The function has a unique global optimum $1^n$ with function value $l = \left\lfloor n/2\right\rfloor +1$. The second best bit string is $0^n$ with function value $l-1$. All other bit strings contain an equal number of 0-blocks and 1-blocks. A bit string with *j* 1-blocks is in level set $L_{j+1}$ and thus, its function value equals $l-(j+1)=l-j-1$.

We defer the proof that MinBlocks is indeed an easiest function for (1+1) CHM to the next section (Theorem 8) in order to make use of arguments from the analysis of the expected optimisation time performed there.

### Expected Optimisation Time

We analyse the expected optimisation time of the (1+1) CHM on MinBlocks, i. e., the expected number of function evaluations executed until the global optimum is reached [12]. To facilitate our analysis, we start with an analysis of the expected optimisation time starting from a bit string from a particular level set which will in turn allow us to prove that MinBlocks is an easiest function for (1+1) CHM. We will then continue with the overall upper and lower bounds for the optimisation time.

#### Lemma 7

We consider (1+1) CHM with strict or non-strict selection on MinBlocks as defined in Definition 6. For $i \in \{0, 1, \dots , l\}$ (where $l+1$ is the number of sets in the partition from Definition 6) let $T_i$ denote the random number of steps needed to reach the unique global optimum $1^n$ when started in a bit string from $L_i$. The expected numbers of steps are ${\text {E}\left( T_0\right) }=0$, ${\text {E}\left( T_1\right) } = n+1$, ${\text {E}\left( T_2\right) } = (n+1)^2/2$ and

$$\begin{aligned} {\text {E}\left( T_i\right) } = \frac{n(n+1)}{(2i-2)(2i-3)} + {\text {E}\left( T_{i-1}\right) } \end{aligned}$$

for all $$i \in \{3, 4, \dots , l\}$$.

#### Proof

The statement about ${\text {E}\left( T_0\right) }$ is trivial. For ${\text {E}\left( T_1\right) }$ it suffices to observe that any hypermutation which chooses as mutation length *n* leads from $0^n$ to the unique global optimum $1^n$. Such a mutation has probability $1/(n+1)$ which implies ${\text {E}\left( T_1\right) } = n+1$. For ${\text {E}\left( T_2\right) }$ we observe that for each $x \in L_2$ there are two mutations which lead to $L_0 \cup L_1$, one leading to $L_0$ and the other leading to $L_1$. Since $L_0$ and $L_1$ are reached with equal probability 1 / 2 we have ${\text {E}\left( T_2\right) } = n(n+1)/2 + {\text {E}\left( T_1\right) }/2 = (n+1)^2/2$. For ${\text {E}\left( T_i\right) }$ with $i>2$ we observe that only mutations that reduce the number of 1-blocks can lead to some $L_j$ with $j<i$. It is easy to see that, the number of 1-blocks can only be reduced by 1 in one contiguous hypermutation. In order to achieve that a mutation must start at the first bit of a block (either a 0-block or a 1-block) and end at the last bit of a block (either a 0-block or a 1-block) but this block cannot be the one just before the block containing the first flipped bit (otherwise the length of the mutation is *n*, the bit string is inverted and the number of 1-blocks remains unchanged). Thus, if there are *j* blocks in the bit string, the number of such mutations equals $j(j-1)$. For $x \in L_i$ the number of 1-blocks equals $i-1$ and therefore the number of blocks equals $2i-2$ so that there are $(2i-2)(2i-3)$ such mutations. Thus, the expected time to leave $L_i$ equals $n(n+1)/((2i-2)(2i-3))$ and ${\text {E}\left( T_i\right) } = n(n+1)/((2i-2)(2i-3))+ {\text {E}\left( T_{i-1}\right) }$ follows. $\square$

The expected runtimes provided in Lemma 7 allow us to verify whether MinBlocks satisfies the criteria set by Lemma 4. The following theorem establishes MinBlocks as an easiest function for (1+1) CHM.

#### Theorem 8

MinBlocks is an easiest function for the (1+1) CHM with strict or non-strict selection among all functions with a unique optimum.

#### Proof

The theorem follows from Lemma 7, the definition of MinBlocks, and Lemma 4. According to Definition 6, for all $i\in \{1, 2, \ldots ,\left\lfloor n/2\right\rfloor \}$ the fitness value of solutions in subset $L_i$ is strictly less than the fitness value of solutions in $L_{i-1}$. Therefore, a solution *x* has a better MinBlocks value than a solution *y*, if and only if *x* and *y* belong to two distinct subsets $L_i$ and $L_j$ respectively such that $i<j$. Note that in Lemma 7 the expected runtime of the (1+1) CHM initialised with a solution from $L_i$, ${\text {E}\left( T_i\right) }$, satisfies ${\text {E}\left( T_i\right) }>{\text {E}\left( T_{i-1}\right) }$ for all *i* and thus $i<j$ implies ${\text {E}\left( T_i\right) }<{\text {E}\left( T_j\right) }$. Therefore, $\textsc {MinBlocks} (x)>\textsc {MinBlocks} (y)$ if and only if the expected runtimes of (1+1) CHM starting from solutions *x* and *y* satisfy $T(A,f,x)={\text {E}\left( T_i\right) } < {\text {E}\left( T_{j}\right) }=T(A,f,y)$. According to Lemma 4, the above two way implication makes MinBlocks an easiest function for the (1+1) CHM.

We continue our analysis of the expected optimisation time. Note that the following bound asymptotically matches the lower bound of ${\varOmega \left( n^2\right) }$ for contiguous hypermutations and functions with a unique global optimum proven by Jansen and Zarges [17].

#### Theorem 9

Let *T* denote the expected optimisation time of the (1+1) CHM with strict or non-strict selection on MinBlocks. ${\text {E}\left( T\right) } = \ln (2) n^2 \pm {O\left( n\right) }$ holds.

#### Proof

Consider $x \in \{0, 1\}^n$ selected uniformly at random. For each ${i {\in } \{0, 1, \dots , n{-}1\}}$ we have that a block ends at *x*[*i*] if $x[i] \not = x[(i+1) \bmod n]$ holds. Thus, *x*[*i*] is the end of a block with probability 1 / 2 and we see that the expected number of blocks equals *n* / 2. Let *I* denote the number of blocks. An application of Chernoff bounds yields that for any constant $\varepsilon$ with $0< \varepsilon < 1$ we have ${\text {Pr}\left( I \ge (1-\varepsilon ) n/2\right) } = 1-e^{-{\varOmega \left( n\right) }}$.

We know that ${\text {E}\left( T \mid I=2(i-1)\right) } = {\text {E}\left( T_i\right) }$ holds and have

$$\begin{aligned} {\text {E}\left( T_i\right) }&= \frac{n(n+1)}{(2i-2)(2i-3)} + {\text {E}\left( T_{i-1}\right) }\\&= \frac{n(n+1)}{(2i-2)(2i-3)} + \frac{n(n+1)}{(2(i-1)-2)(2(i-1)-3)} + {\text {E}\left( T_{i-2}\right) }\\&= \cdots = {\text {E}\left( T_2\right) } + n(n+1) \sum _{j=0}^{i-3} \left[ \frac{1}{(2(i-j)-2)(2(i-j)-3)} \right] \\&= {\text {E}\left( T_2\right) } + n(n+1) \sum _{j=0}^{i-3} \left[ \frac{1}{2j+3} + \frac{1}{2j+4} - \frac{1}{j+2} \right] \\&= {\text {E}\left( T_2\right) } + n(n+1) \left( \left( \sum _{j=3}^{2i-2} \frac{1}{j} \right) - \left( \sum _{j=2}^{i-1} \frac{1}{j} \right) \right) \\&= {\text {E}\left( T_2\right) } + n(n+1) \left( H_{2i-2} - H_{i-1} - \frac{1}{2} \right) \end{aligned}$$

where $H_j$ denotes the $j^{\text {th}}$ harmonic number. Using $H_j = \ln (j) + \gamma + 1/(2j) - {o\left( 1/j\right) }$ ($\gamma = 0.57721\dots$ the Euler-Mascheroni constant) we obtain

$$\begin{aligned} {\text {E}\left( T \mid I=2(i-1)\right) }&= \frac{(n+1)^2}{2} + n(n+1) \left( \ln (2) - \frac{1}{2} \right) - {O\left( n^2/i\right) } \\&= \ln (2) n^2 + {O\left( n\right) } - {O\left( n^2/i\right) }. \end{aligned}$$

For the lower bound on ${\text {E}\left( T\right) }$ we use $i=(n/8)+1$ (and have ${\text {Pr}\left( I \ge n/4\right) } = 1-e^{-{\varOmega \left( n\right) }}$, of course) and obtain

$$\begin{aligned} {\text {E}\left( T\right) }&\ge {\text {Pr}\left( I \ge n/4\right) } \cdot {\text {E}\left( T \mid I \ge n/4\right) } \\&= \left( 1-e^{-{\varOmega \left( n\right) }}\right) \cdot \left( \ln (2) n^2 + {O\left( n\right) } - {O\left( n\right) } \right) = \ln (2) n^2 \pm {O\left( n\right) } \end{aligned}$$

as claimed.

For the upper bound on ${\text {E}\left( T\right) }$ we have

$$\begin{aligned} {\text {E}\left( T\right) } \le {\text {E}\left( T_{n/2}\right) } = \ln (2) n^2 + {O\left( n\right) } - {O\left( n\right) } = \ln (2) n^2 \pm {O\left( n\right) } \end{aligned}$$

as claimed. $$\square $$

### Fixed Budget Analysis

It has been pointed out that the notion of optimisation time does not always capture the nature of how randomised search heuristics are applied in practice. As a result, fixed budget analysis has been introduced as an alternative theoretical perspective [19, 20]. Let $x_t$ denote the current population after *t* rounds of contiguous hypermutation and selection. In fixed budget analysis we want to analyse ${\text {E}\left( f(x_t)\right) }$ for all $t \le {\text {E}\left( T\right) }$ where ${\text {E}\left( T\right) }$ is the expected optimisation time. We do this here for MinBlocks to give a more complete picture about the performance of the (1+1) CHM. Note that a comparison of the (1+1) EA and the (1+1) CHM under the fixed budget perspective has previously been performed for some example functions [21].

We begin with a statement about the expected function value of a uniform random solution, reflecting the way (1+1) $A$ algorithms are initialised, and prove that it is roughly $(n-2)/4 \pm 1/2$.

#### Theorem 10

Let $x_0 \in \{0, 1\}^n$ be selected uniformly at random and $f := \textsc {MinBlocks}$ from Definition 6. For the initial function value ${\text {E}\left( f(x_0)\right) } = \left\lfloor n/2\right\rfloor -(n/4)+2^{-n}$ holds.

#### Proof

We know from the analysis of the expected optimisation time in Sect. 4.2 that the expected number of blocks in $x_0$ equals *n* / 2. Let $z(x_0)$ denote the number of 0-blocks in $x_0$ and remember that the number of 0-blocks is half the number of blocks. This implies ${\text {E}\left( z(x_0)\right) } = n/4$.

Let $l=\left\lfloor n/2\right\rfloor +1$ (the value defined in Definition 6). Remember that $z(1^n) = z(1^0) = 0$ according to our definition of a 0-block (see the definition of the notation at the beginning of Sect. 4.1). For $x_0 \notin \{ 0^n, 1^n \}$ we have $f(x_0) = l-1-z(x)$. Furthermore, we have $f(1^n)=l$ and $f(0^n) = l-1 = l-1-z(0^n)$. Thus, $f(x_0) = l-1-z(x)$ holds always except for the case $x_0 = 1^n$.

We have

$$\begin{aligned} {\text {E}\left( f(x_0)\right) }&= \sum \limits _{x \in \{0, 1\}^n} \frac{f(x)}{2^n} = \sum \limits _{x \in \{0, 1\}^n \setminus \{ 1^n \}} \left[ \frac{l-1-z(x)}{2^n} \right] + \frac{l}{2^n} \\&= \sum \limits _{x \in \{0, 1\}^n \setminus \{ 1^n \}} \left[ \frac{l-1-z(x)}{2^n} \right] + \frac{l}{2^n} + \frac{l-1-z(1^n)}{2^n} - \frac{l-1-z(1^n)}{2^n} \\&= \sum \limits _{x \in \{0, 1\}^n} \left[ \frac{l-1-z(x)}{2^n} \right] + \frac{l}{2^n} - \frac{l-1-z(1^n)}{2^n} \\&= \sum \limits _{x \in \{0, 1\}^n} \left[ \frac{l-1-z(x)}{2^n} \right] + \frac{1}{2^n} = {\text {E}\left( l-1-z(x_0)\right) } + \frac{1}{2^n} \\&= l-1-{\text {E}\left( z(x_0)\right) } + 2^{-n} = l-1-(n/4)+2^{-n} \end{aligned}$$

and obtain the claimed bound by remembering that $l = \left\lfloor n/2\right\rfloor +1$ holds. $\square$

We now give upper and lower bounds on the expected function value after *t* iterations of the (1+1) CHM.

#### Theorem 11

Let $x_t \in \{0, 1\}^n$ denote the current search point after random initialisation and *t* rounds of contiguous hypermutation and strict or non-strict selection on $f := \textsc {MinBlocks}$. The following bounds hold for the expected function value after *t* steps ${\text {E}\left( f(x_t)\right) }$.

$$\begin{aligned} \mathbf{lower bound }\;&{\text {E}\left( f(x_0)\right) } + \left( \left\lfloor \frac{n}{2}\right\rfloor +1 - {\text {E}\left( f(x_0)\right) }\right) \left( 1-\left( 1-\frac{2}{n(n+1)}\right) ^t\right) \\&\le \left\lfloor \frac{n}{2}\right\rfloor +1 - \left( \left\lfloor \frac{n}{2}\right\rfloor +1-{\text {E}\left( f(x_0)\right) }\right) \cdot \left( 1-\frac{3}{n(n+1)}\right) ^t \\&\le {\text {E}\left( f(x_t)\right) } \\ \mathbf{upper bound }\;&{\text {E}\left( f(x_t)\right) } \\&\le \sum \limits _{z=1}^{n/2} \left[ \left( {\begin{array}{c}n/2\\ z\end{array}}\right) 2^{-n/2} \cdot \Bigg ( \left( \frac{n}{2} - z \right) + 1 - \left( 1-\frac{1}{n+1}\right) ^t \right. \\&\quad \left. + \sum \limits _{d=2}^{z+1} \left[ 1 - \left( 1 - \frac{(2d-2) (2d-3)}{n(n+1)}\right) ^t \right] \Bigg ) \right] + n \cdot 2^{-n+1} \end{aligned}$$

#### Proof

The function value can increase at most $\left\lfloor n/2\right\rfloor +1 - z$ times if the initial function value is *z* since the maximal function value is $\left\lfloor n/2\right\rfloor +1$. For an upper bound we consider the actual probabilities for increasing the function value which equal $(2d-2)(2d-3)/(n(n+1))$ if the current number of 0-blocks is *d* and it is ${1/(n+1)}$ for $0^n$. For each of these events we add the expected contribution after *t* steps. This contribution equals the probability to have at least one such increasing step in *t* steps which equals ${1-(1-p)^t}$ if *p* denotes the probability for an increasing step. Adding these yields an upper bound since it pretends that for each level *t* steps are available to create an increasing step whereas in reality decreasing the number of 0-blocks from *d* to $d-1$ is only possible after it has been decreased to *d* from $d+1$ before. Since initialisation in $0^n$ or $1^n$ has probability $2^{-n+1}$ the contribution of these cases is ${O\left( n/2^n\right) }$. Let *Z* denote the random number of 0-blocks in the initial bit string. We obtain

$$\begin{aligned} {\text {E}\left( f(x_t)\right) } \le&\sum \limits _{z=1}^{n/2} \Bigg [ {\text {Pr}\left( Z=z\right) } \cdot \Bigg ( \left( l - (z+1) \right) \\&+ 1 - \left( 1-\frac{1}{n+1}\right) ^t + \sum \limits _{d=2}^{z+1} \left[ 1 - \left( 1 - \frac{(2d-2) (2d-3)}{n(n+1)}\right) ^t \right] \Bigg ) \Bigg ] \\&+ l \cdot 2^{-n+1} \end{aligned}$$

using the law of total probability. We obtain an upper bound by noting that *Z* is binomially distributed with parameters *n* / 2 and 1 / 2, and by remembering that $l = \left\lfloor n/2\right\rfloor +1$ holds.

For the smaller of the two lower bounds we replace the actual probabilities by the smallest probability for an increase in function value which equals $2/(n(n+1))$. We can improve on this weak lower bound slightly by using a technique called multiplicative fixed budget drift as recently introduced by Lengler and Spooner [26]. For a lower bound we need a lower bound on the expected change in function value in one generation given the current function value. If the current bit string contains *i* bits, we know that the expected change equals $(2i-2)(3i-2)/(n(n+1))> 3i^2/(n(n+1)) > 3i/(n(n+1))$ where the last inequality is made because Theorem 1 in [26] requires a statement about this drift that is linear. Using this we obtain $\left\lfloor n/2\right\rfloor +1 - \left( \left\lfloor n/2\right\rfloor +1-{\text {E}\left( f(x_0)\right) }\right) \cdot (1-3/(n(n+1)))^t$ as lower bound on the expected function value after *t* steps.

**Fig. 1:**
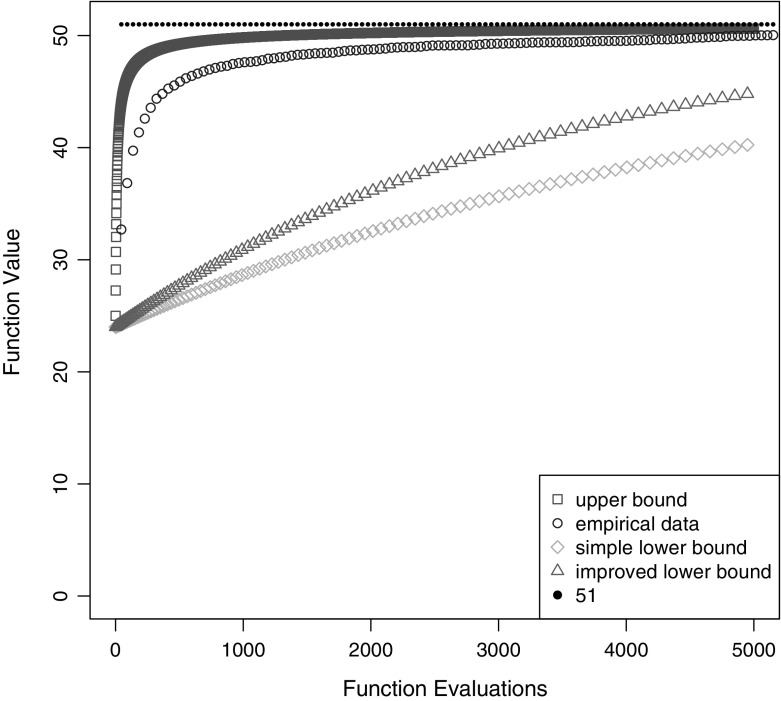
Average function values for the (1+1) CHM on an easiest function for the (1+1) CHM with unique global optimum for $n=100$ after a given number of function evaluations, averaged over 100 runs, together with the theoretical bounds from Theorem 11. The empirical data was produced with a (1+1) CHM with strict selection

To obtain actual lower and upper bounds we use the lower and upper bounds for the initial expected function value from Theorem 10. While the bounds from Theorem 11 are not simple and in particular the upper bound is not even a closed form, we can easily evaluate them numerically for reasonable values of *n*. In Fig. 1 we display them for $n=100$ together with the maximal function value 51 and empirical results averaged over 100 runs. We see that while the upper bound yields reasonable results both lower bounds are rather weak.

## Hybridising Operators

A popular approach in areas such as memetic algorithms [27] or hyper-heuristics [30] is to combine several operators in one algorithm. There are many ways of hybridising algorithms – here, we consider four different schemes of hybridisation that combine *k* operators. Given several (1+1) $A$ s, $A_1, \cdots , A_k$, the different hybridisations considered are:

**(1+1) HA**: executing one operator chosen probabilistically according to a given probability distribution ${\varvec{p}}= (p_1, \dots , p_k)$ (Algorithm 4),
**(1+1) HA-chained**: executing a chain of operators, where operators are applied probabilistically according to a given probability vector ${\varvec{p}}= (p_1, \dots , p_k)$ (Algorithm 5, note that ${\varvec{p}}$ does not have to be a probability distribution),
**(1+*k*) HA**: executing all operators in parallel and picking the best among the resulting solutions (Algorithm 6), and
$${{\varvec{k}}} \times \mathbf (1+1)~HA $$:
executing all operators sequentially, resulting in a chained sequence of operations, with selection after each operator (Algorithm 7).

The (1+1) HA-chained is probably the most commonly used method in hybrid algorithms. For instance, most Genetic Algorithms fall into this category since they use a probability $p_c$ that crossover is applied before mutation. The (1+1) HA is commonly used in memetic algorithms and hyper-heuristics when at each step a variation operator is chosen with some probability; this strategy was called “SimpleRandom” in [5]. Theoretical work on simple hyper-heuristics that fall into this framework have previously been accomplished [24]. We also introduce two other hybridisation schemes for which the results presented in this section also hold. These are simplified versions of schemes that are applied in widely used hybrid algorithms. The (1+*k*) HA algorithm creates offspring by using different operators. This is a common strategy in multi-meme memetic algorithms, where multiple memes refer to multiple local improvement heuristics. The idea was called “Greedy approach” in [5]. The same idea also appears in simple island models with heterogeneous islands, that is, each island consists of one individual and uses a different variation operator. A real-world example of where such a strategy is used (albeit with populations and added complexity) is the Wegener system, a popular and effective algorithm in search-based software testing, where different islands use standard bit mutations with different mutation rates [36]. The $k \times$(1+1) HA is interesting as it has some resemblance to the recently introduced (1+($\lambda$,$\lambda$)) GA, since both use selection between the application of the operators. However, the (1+($\lambda$,$\lambda$)) GA does not fall exactly within the $k \times$(1+1) HA scheme. In the former, if the final solution is worse than the initial one in the sequence, then the initial solution is accepted for the next generation. On the other hand, the $k \times$(1+1) HA always accepts the last improving solution in the sequence.

Note that (1+1) HA-chained and $k \times$(1+1) HA differ in the use of selection: the latter applies selection after each operator, whereas the former only applies selection at the end of the generation. One generation of (1+*k*) HA may be regarded as a derandomised version of (1+1) HA$(1/k, \dots , 1/k)$ run for *k* generations. In the former all operators are executed once, whereas in the latter algorithm all operators are executed once *in expectation*. The latter also admits non-uniform probabilities.
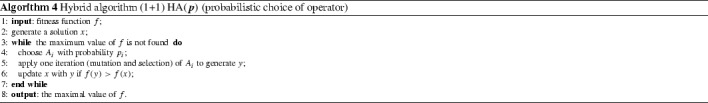
 
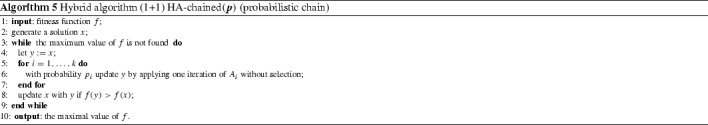
 
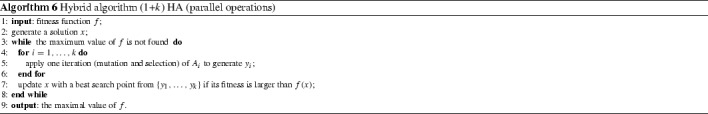
 
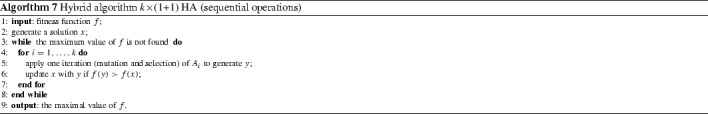


The B-cell algorithm (BCA) [23] is an example of an algorithm that uses both SBM and CHM considered in this paper. More specifically, it uses a population of search points and creates $\lambda$ clones for each of them. It then applies standard bit mutation to a randomly selected clone for each parent search point and subsequently applies CHM to all clones. This way one offspring of each parent is subject to a sequence of two mutations, first standard bit mutation and afterwards CHM. Jansen et al. [14] proposed a variant of the BCA that only uses CHM with constant probability $0<p<1$ (instead of $p=1$) and were able to show significantly improved upper bounds on the optimisation time for this algorithm on instances of the vertex cover problem. Considering the individuals that undergo both kinds of mutation (or a (1+1)-style BCA), both these variants fit within the (1+1) HA-chained model of hybridisation (Algorithm 5). More precisely, for ${\varvec{p}}= (p_1, p_2)$ with $p_1$ the probability to execute SBM and $p_2$ the probability to execute CHM, we have $p_1=p_2=1$ for the original BCA and constant $p_1=1$ and $0< p_2 < 1$ for the modified BCA in [14]. We remark that the improved results in [14] in fact hold as long as $p_1 = {\varOmega \left( 1\right) }$.

In the following subsection we will first consider the general hybrid algorithmic framework and then specialise the results to the combination of CHM and SBM.

### The Advantage of Hybridisation

The easiest functions for SBM and CHM are OneMax and MinBlocks, respectively. Before analysing hybrid algorithms using both operators, it is natural to consider the effect of one operator on the easiest function for the other operator. It is well known that the (1+1) CHM needs $\Theta (n^2 \log n)$ expected time on OneMax  [17, 21].

Here we consider the expected optimisation time of the (1+1) EA on MinBlocks and show that it is very inefficient.

#### Theorem 12

The expected optimisation time of the (1+1) EA with strict or non-strict selection on MinBlocks is at least $n^n/2$.

#### Proof

The function MinBlocks has the following property: for all search points except for $0^n$ and $1^n$, the number of 0-blocks equals the number of 1-blocks. Notice that inverting all bits in a bit string turns all 0-blocks into 1-blocks and vice versa. Hence for all $x \notin \{0^n, 1^n\}$ we have $\textsc {MinBlocks} (x) = \textsc {MinBlocks} (\overline{x})$.

Let $x_0, x_1, \dots$ be the trajectory of the (1+1) EA and $T \in {\mathbb {N}}_0$ be the first hitting time of a search point in $\{0^n, 1^n\}$. Since $x_0, x_1, \dots , x_T$ has the same probability as $\overline{x_0}, \overline{x_1}, \dots , \overline{x_T}$ and $x_T \in \{0^n, 1^n\}$, we have ${\text {Pr}\left( x_T = 0^n\right) } = 1/2$. In this case the only accepted search point is the global optimum $1^n$, for which all bits have to be flipped in one mutation. This has probability $n^{-n}$ and expected waiting time $n^n$. Combined with the probability of reaching this state, the expected optimisation time is at least $T + n^n/2 \ge n^n/2$. $\square$

Note that the expected optimisation time of the (1+1) EA on MinBlocks is only by a factor of at most 2 smaller than the expected optimisation time of the (1+1) EA on its hardest function, Trap, which is almost $n^n$ [9].

Using multiple operators, the hope is that the advantages of each operator are combined. However, this is not always true: new operators can make a hybrid algorithm follow an entirely different search trajectory and lead to drastically increased optimisation times. This behaviour was demonstrated for memetic algorithms [31] as well as for standard bit mutations cycling between different mutation rates [15] and for population based EAs where the mutation rate of each individual depends on its rank in the population [28].

We show that such effects cannot occur when dealing with easiest functions. If *f* is an easiest function for *A* which is a (1+1) $A$, then Theorem 13 stated below allows to transfer an upper bound on the expected optimisation time of *A* to the four hybrid algorithms.

#### Theorem 13

If $A_1, \dots , A_k$ are (1+1) $A$ ’s with strict or non-strict selection, starting in $x_0$, and *f* is an easiest function for $A_i$, then the expected hitting time of (1+1) HA$({\varvec{p}})$ on *f*, for a probability distribution ${\varvec{p}}= (p_1, \dots , p_k)$, is bounded from above by

$$\begin{aligned} \frac{1}{p_i} \cdot T(A_i, f, x_0). \end{aligned}$$

For any probability vector ${\varvec{p}}= (p_1, \dots , p_k)$ with $p_i > 0$ and $p_j < 1$ for all $j \ne i$, the expected hitting time of (1+1) HA-chained$({\varvec{p}})$ on *f* is bounded from above by

$$\begin{aligned} \frac{1}{p_i \cdot \prod _{j\ne i} (1-p_j)} \cdot T(A_i, f, x_0). \end{aligned}$$

Moreover, the expected hitting time of (1+*k*) HA and $k \times$(1+1) HA is bounded from above by

$$\begin{aligned} k \cdot T(A_i, f, x_0). \end{aligned}$$

#### Proof

We follow the analysis in [10, Section III] and perform a drift analysis, choosing the runtime $T(A_i, f, x)$ as the drift function:

$$\begin{aligned} d(x)= T(A_i, f, x). \end{aligned}$$

Define $\varDelta _{A_j}(x) = {\text {E}\left( (d(x)-d(y))\right) }$ as the drift of Algorithm $A_j$, given that *y* was created by applying $A_j$ to *x*. The drift of the hybrid algorithm (1+1) HA$({\varvec{p}})$ is

$$\begin{aligned} \varDelta _{(1+1)~HA} (x) = \sum _{i=1}^k p_i \varDelta _{A_i}(x). \end{aligned}$$

We apply Lemma 3 in [10] to estimate $\varDelta _{A_i}$. For any non-optimal point *x*, let *y* be its child, then the drift of algorithm $A_i$ satisfies

3 $$\begin{aligned} \varDelta _{A_i}(x) = E {(d(x)-d(y))} =1 \end{aligned}$$

following from the definition of $d(x) = T(A_i, f, x)$. We further claim that no operator induces a negative drift. Given any two non-optimal points *x* and *y*, then when using strict selection, according to the monotonically decreasing condition $d(x) < d(y)$ implies $f(x) > f(y)$. By contraposition, we get

4 $$\begin{aligned} f(y) \ge f(x) \Rightarrow d(y) \le d(x). \end{aligned}$$

When using non-strict selection, the strictly monotonically decreasing condition implies $f(y) \ge f(x) \Leftrightarrow d(y) \le d(x)$, which implies (4) as well. Since all algorithms $A_1, \dots , A_k$ adopt elitist selection, the distance cannot increase, regardless of which operator is chosen. Hence $\varDelta _{A_j}(x) \ge 0$ for all *j* and

$$\begin{aligned} \varDelta _{(1+1)~HA} (x) \ge p_i \varDelta _{A_i}(x) = p_i. \end{aligned}$$

Using the additive drift theorem, Theorem 2, the expected hitting time of (1+1) HA$({\varvec{p}})$ on *f* is at most

$$\begin{aligned} \frac{d(x_0)}{p_i} = \frac{T(A_i, f, x_0)}{p_i}. \end{aligned}$$

For (1+1) HA-chained we observe that the algorithm executes only $A_i$ and none of the other operators with probability $p_i \cdot \prod _{j \ne i} (1-p_j)$. In all other cases the distance cannot increase (by (4)). Hence by the same arguments as above, the expected hitting time is bounded by

$$\begin{aligned} \frac{1}{p_i \cdot \prod _{j\ne i} (1-p_j)} \cdot T(A_i, f, x_0). \end{aligned}$$

The statement on $(1+k)$ HA follows from similar arguments. Let $x_1, \dots , x_k$ be the search points created using $A_1, \dots , A_k$, respectively. Let $x^*$ be the best amongst these, selected for survival. Then $f(x^*) \ge f(x_i)$, and by (4), $d(x^*) \le d(x_i)$. Hence for all non-optimal *x*,

$$\begin{aligned} \varDelta _{(1+k)~HA}(x) \ge \varDelta _{A_i}(x) = 1. \end{aligned}$$

Additive drift from Theorem 2 then yields an upper bound on the expected time of (1+*k*) HA of $k \cdot T(A_i, f, x_0)$, the factor *k* accounting for executing *k* operations in one generation.

Finally, for $k \times$(1+1) HA, let $x_1, \dots , x_k$ be the offspring created in the sequence of operations and note that $x_k$ is taken over for the next generation. We have $f(x_{i-1}) \ge \dots \ge f(x_1) \ge f(x)$ and thus $d(x_{i-1}) \le d(x)$ by (4). Along with ${\varDelta _{A_i}(x) = 1}$ for all non-optimal *x* and $f(x_k) \ge f(x_i)$ implying $d(x_k) \le d(x_i)$, we get

$$\begin{aligned} \varDelta _{k \times (1+1)~HA}(x) \ge \varDelta _{A_i}(x) = 1 \end{aligned}$$

and an upper bound of $k \cdot T(A_i, f, x_0)$ as for (1+*k*) HA. $\square$

Using Theorem 13 as well as Theorem 9, we get the following corollary concerning the SBM and CHM operators.

#### Corollary 14

Consider the hybrid algorithms (1+1) HA$({\varvec{p}})$ and (1+1) HA-chained$({\varvec{p}})$ for ${\varvec{p}}= (p_1, p_2)$ with constant $p_1, p_2 > 0$, (1+2) HA, and $2 \times$(1+1) HA, all based on a (1+1) algorithm $A_1$ using SBM and another (1+1) algorithm $A_2$ using CHM; $A_1$ and $A_2$ both using strict or non-strict selection. Then the expected optimisation time of all these hybrids on OneMax and MinBlocks is $O(n \log n)$ and $O(n^2)$, respectively.

All hybrid algorithms are hence able to combine the advantages of both operators on the two easiest functions for its two operators. This is particularly true for the modified (1+1)-style BCA [14] with constant $0< p_1, p_2 < 1$ discussed at the beginning of Sect. 5.

### Weighted Combinations of OneMax and MinBlocks

In the previous subsection it was proven that the four different hybrid schemes (1+1) HA$({\varvec{p}})$ for ${\varvec{p}}= (p_1, p_2)$ with constant $p_1, p_2 > 0$, (1+1) HA-chained, (1+2) HA, and $2 \times$(1+1) HA, using SBM and CHM as operators are all efficient for OneMax and MinBlocks. In particular, even though SBMs alone exhibit very poor performance on MinBlocks, hybrid algorithms using CHM with arbitrarily low constant probability along with SBM are efficient. In this subsection we investigate the performance of the two operators on “hybrid” functions where the fitness depends on both the number of ones and the number of blocks.

To this end, we perform experiments concentrating on different instantiations of Algorithm (1+1) HA$({\varvec{p}})$ for ${\varvec{p}}= (p, 1-p)$ with various values of *p* and strict selection and investigate its performance on a function consisting of weighted combinations of OneMax and MinBlocks. To be more precise we consider the function

$$\begin{aligned} f_w(x)=w \cdot \text {OneMax} (x) +(1-w) \cdot \textsc {MinBlocks} (x) \end{aligned}$$

with $w\in \{0, 0.1, 0.2, \ldots , 1.0 \} \cup \{1/n, 1/n^2\}$ and the (1+1) HA$(p,1-p)$ where we execute CHM with probability *p* and standard bit mutations with probability $1-p$ with $p\in \{0, 0.1, 0.2, \ldots , 1.0\} \cup \{1/n, 1/n^2\}$. Note, that for $p=0$ and $w=0$ we only use standard bit mutations on pure MinBlocks and thus, the optimisation time is exponential (Theorem 12). Similarly, we have an optimisation time of ${\Theta \left( n \log n\right) }$ for $p=0$ and $w=1$ (standard bit mutations on pure OneMax [9]), ${\Theta \left( n^2\log n\right) }$ for $p=1$ and $w=1$ (CHM on pure OneMax [17]) and ${\Theta \left( n^2\right) }$ for $p=1$ and $w=0$ (CHM on pure MinBlocks, Theorem 9). We are particularly interested in intermediate values of *p* and *w* and their influence on the optimisation time.

**Fig. 2:**
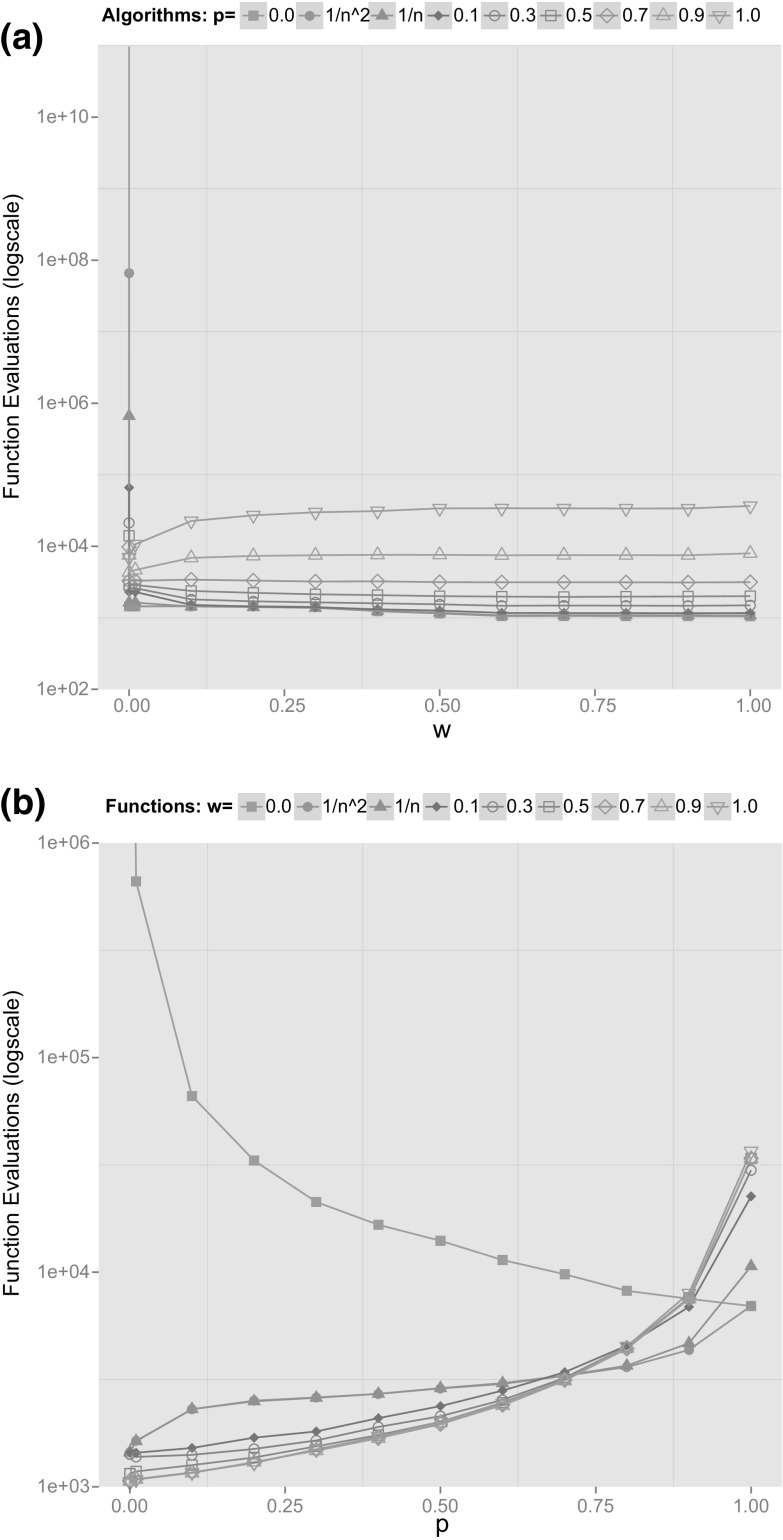
Results of the experiments: Average optimisation times over 10,000 independent runs for each pair of *p* and *w* for $n=100$. The empirical data was produced with a (1+1) HA$(p,1-p)$ with strict selection. **a** Results for different algorithms (1+1) HA$(p,1-p)$ over values for *w*. **b** Results for different functions $f_w$ over values for *p*

We perform 10,000 runs for each of the above pairs of settings for $n=100$ and depict the average optimisation times in Fig. 2. Figure 2a shows a comparison of the optimisation times of different resulting algorithms (1+1) HA$(p,1-p)$ depending on *w* while Fig. 2b depicts the results for different functions over the parameter *p* of the hybrid algorithm. Note, that for the sake of recognisability we only depict a subset of the curves in both figures while on each curve we present all available data points. We additionally perform Wilcoxon signed rank tests to assess whether the observed differences in the optimisation times are statistically significant. Fixing a value for *w* (Fig. 2a) we perform tests for all pairs of algorithms and the 10,000 optimisation times measured for each setting. Similarly, fixing a value for *p* (Fig. 2b) we perform tests for all pairs of functions. We perform Holm-Bonferroni correction to account for the large number of tests we execute.

We observe that for all $w > 0$, the runtime gets smaller as *p* decreases (Fig. 2a). All differences are statistically significant at confidence level 0.05 with the exception of $p=0$ and $p=1/n^2$ for all values of *w* and $p=0$, $p=1/n^2$, $p=1/n$ and $p=0.1$ for most $w \in \{0.1, 0.2, 0.3, 0.4\}$. From Fig. 2b we can also see that the (1+1) HA$({\varvec{p}})$ with constant $p < 0.7$ appears to be faster on functions $f_w$ with constant $w>0$ (i. e., with constant OneMax fraction) while using a larger *p* (i. e., performing CHM more often) pays off for $w={o\left( 1\right) }$. Differences are statistically significant at confidence level 0.05 with the exception of most results for $p=0.7$ and $p=0.8$, $w=1/n$ and $w=1/n^2$ for all $p \le 0.8$, $w \ge 0.6$ for all *p*, $w \ge 0.3$ for all $p \ge 0.5$, and $w \le 0.2$ for $w=0$, $w=1/n^2$ and $w=1/n$.

We can see from the experiments in Fig. 2 that the case $w=0$ is very different from runs with $w>0$. Recall that $w=0$ means that the algorithm is confronted with $\textsc {MinBlocks}$ and that $w>0$ implies that we add $w \cdot \text {OneMax}$ to the function value (while, at the same time, reducing the function value by $w \cdot \textsc {MinBlocks}$). The addition of $w \cdot \text {OneMax}$ with an arbitrarily small $w>0$ introduces a ‘search gradient’ in the fitness landscape: the algorithm is encouraged to increase the number of 1-bits by a small increase in fitness (at least as long as this trend is not counteracted by an increase in the number of blocks). The effect is most pronounced for $p=0$, i. e., for the (1+1) EA where the expected optimisation time is exponential for $\textsc {MinBlocks}$ and becomes manageable as soon as the $\text {OneMax}$-component introduces a ‘search gradient’. In a different context, the same effect has been discussed and analysed using different example functions [16].

### On the Easiest Function for Hybrid Algorithms

In the previous subsection it was shown experimentally that as soon as a small OneMax component comes into play, the function $f_w$ becomes easy for the (1+1) HA(*p*,$1-p$) using SBM and CHM independent of the value of *p*. Nevertheless, in this subsection we show that $f_w$, for any value of *w*, is not an easiest function for the (1+1) HA(*p*,$1-p$). In particular, we will set $p=1/2$ and show that the easiest function for the hybrid algorithm (1+1) HA(1 / 2,1 / 2) using SBM and CHM, and strict selection, is more complex than a mere weighted combination $f_w$ of the two easiest functions for both operators.

He et al. [10] explain how an easiest function can be computed. We construct $\textsc {EasiestHybrid}_p$, an easiest fitness function for the hybrid algorithm (1+1) HA(*p*,$1-p$) from Corollary 14 by implementing their construction procedure and performing the necessary computations numerically. Clearly, this is computationally feasible only for small values of *n*. Using the unique global optimum as a starting point and level $L_0$ we can compute the next level of search points with next best and equal fitness by computing for each search point which does not yet have a level the expected time needed to reach $L_0$ either directly or via a mutation to a search point that already has a level. Search points with minimal time in this round make up the next level. Note that this is actually Dijkstra’s algorithm for computing shortest paths (see, e. g., [3]). Also note that the numerical computation of the actual expected waiting times is easy since the exact transition probabilities for mutations leading from one bit string to another are all known and waiting times are all simply geometrically distributed.

The easiest function for the (1+1) CHM is composed of $\left\lfloor n/2\right\rfloor +2$ different fitness levels, $L_0, \dots , L_{\left\lfloor n/2\right\rfloor +1}$, defined by a number of 1-blocks (i. e., $L_0 = \{1^n\}$, $L_1 = \{0^n\}$, $L_i = \{x \in \{0,1\}^n \mid x$ contains $i-1$ different 1-blocks$\}$ for each $i \in \{2, \dots , \left\lfloor n/2\right\rfloor +1\}$). On the other hand, the fitness level set of the easiest function for the (1+1) EA (i. e., OneMax) has $n+1$ different levels defined by a number of 1-bits (i. e., $L_i = \{x \in \{0,1\}^n \mid x$ contains $n- i$ 1-bits$\}$ for each $i \in \{0,\dots , n\}$). If $\textsc {EasiestHybrid}_p$ was a mere weighted combination of OneMax and MinBlocks, its fitness levels would be defined by a combination of a number of 1-blocks and a number of 1-bits. This would happen because individuals that have the same number of 1-bits and the same number of 1-blocks would have exactly the same fitness. Also, the product between the number of levels of the easiest functions for each operator, $(\left\lfloor n/2\right\rfloor +2) \cdot (n+1)$, would be an upper bound on the number of levels for $\textsc {EasiestHybrid}_p$. In the following we show that neither of these two considerations are true and that the fitness levels of the easiest function for the hybrid algorithm are more complicated. In particular, bit strings having in common the same number of 1-blocks and the same number of 1-bits can belong to different fitness levels of $\textsc {EasiestHybrid}_p$. Rather, the *length of the blocks* comes into play to define the fitness levels even though such a feature does not define the levels of either OneMax or MinBlocks.

**Table 1:** Fitness level landscape of the $\textsc {EasiestHybrid}_{1/2}$ function for $n=6$

**Level**	**Definition**	**Size**	**Example**	$\mathbf {P_{L_7}}$	$\mathbf {P_{L_8}}$
$L_0$	|1-blocks| = 0, $x=\{1^n\}$	1	111111	=	=
$L_1$	|1-blocks| = 0, $x=\{0^n\}$	1	000000	=	=
$L_2$	|1-blocks| = 1; length = {5}	6	111110	$1/2 \cdot (\mathbf {3} P_{\mathrm {CHM}} + 2 P_{\mathrm {SBM}_1} + 4 P_{\mathrm {SBM}_3})$	$1/2 \cdot (\mathbf {2} P_{\mathrm {CHM}} + 2 P_{\mathrm {SBM}_1} + 4 P_{\mathrm {SBM}_3})$
$L_3$	|0-blocks| = 1; length = {5}	6	000001	$1/2 \cdot (\mathbf {3} P_{\mathrm {CHM}} + 4 P_{\mathrm {SBM}_3} + 2 P_{\mathrm {SBM}_5})$	$1/2 \cdot (\mathbf {2} P_{\mathrm {CHM}} + 4 P_{\mathrm {SBM}_3} + 2 P_{\mathrm {SBM}_5})$
$L_4$	|1-blocks| = 1; length = {4}	6	111100	=	=
$L_5$	|0-blocks| = 1; length = {4}	6	000011	=	=
$L_6$	|1-blocks| = 1; length = {3}	6	111000	$1/2 \cdot (\mathbf {2} P_{\mathrm {CHM}} + \mathbf {1} P_{\mathrm {SBM}_1} + 4 P_{\mathrm {SBM}_3} + 1 P_{\mathrm {SBM}_5})$	$1/2 \cdot (\mathbf {4} P_{\mathrm {CHM}} + 6 P_{\mathrm {SBM}_3})$
$L_7$	|1-blocks| = 2; length = {3,1}	6	111010		
$L_8$	|1-blocks| = 2; length = {2,2}	3	110110		
$L_9$	|0-blocks| = 2; length = {3,1}	6	000101		
$L_{10}$	|1-blocks| = 2; length = {2,1}	12	110100		
$L_{11}$	|0-blocks| = 2; length = {2,2}	3	001001		
$L_{12}$	|1-blocks| = 3; length = {1,1,1}	2	101010		

Columns $P_{L_7}$ and $P_{L_8}$ report the probabilities of reaching levels of higher fitness respectively from levels $L_7$ and $L_8$. Values are only reported when $P_{L_7,L_i} \ne P_{L_8,L_i}$ for $i \in \{0,\dots 6\}$. $P_{\mathrm {CHM}}=1/(6 \cdot 7)$ is the probability of one specific CHM and $P_{\mathrm {SBM}_i}=(1/6)^i\cdot (5/6)^{6-i}$ is the probability of a specific SBM flipping *i* bits

We consider Algorithm (1+1) HA(*p*,$1-p$) which at each step executes either an SBM or a CHM with probability $p=1/2$ and report in Table 1 the different fitness levels $L_0,\dots , L_{12}$ of $\textsc {EasiestHybrid}_{1/2}$ when $n=6$. From the table it can be noticed that both levels $L_7$ and $L_8$ contain bit strings with two 1-blocks and four 1-bits. However, the lengths of the two 1-blocks are different (i. e., three and one for $L_7$ and two and two for $L_8$). The reason why levels $L_7$ and $L_8$ are distinct is highlighted in the last two columns of Table 1, which report the probabilities of reaching levels of higher fitness, respectively from levels $L_7$ and $L_8$. For any point of the search space belonging to level $L_7$ there are three different CHMs leading to points in $L_2$ and $L_3$ while there are only two CHMs from points in $L_8$. These extra CHMs lead to higher transition probabilities, hence lower runtimes, to reach levels $L_2$ and $L_3$ from $L_7$, compared to $L_8$. Also the probability of reaching level $L_6$ is higher from $L_7$, mainly because the level may be reached by flipping only one bit while at least three bits need to be flipped for bit strings in $L_8$. Since the transition probabilities to the remaining levels of higher fitness are the same, the expected runtime from $L_7$ is lower than that from $L_8$, explaining why the two levels are distinct (recall that, by construction, the levels are ordered according to increasing expected runtimes).

Concerning the number of different fitness levels, these increase as the problem size *n* increases. Already for $n=10$ $\textsc {EasiestHybrid}_{1/2}$ has 78 different levels, more than the product of the number of OneMax and MinBlocks levels for $n=10$ (i. e. 66). It is indeed the increase in number of fitness levels as *n* grows that makes it hard to give a precise definition of the $\textsc {EasiestHybrid}_{1/2}$ function. We leave this as an open problem for future work.

## Conclusions

We have extended the analysis of easiest function classes from standard bit mutations to the contiguous somatic hypermutation (CHM) operator used in artificial immune systems. Albeit the recent advances in their theoretical foundations [21, 29, 35] no such results were available concerning artificial immune system operators. With the runtime and fixed budget analyses of the (1+1) CHM on MinBlocks, the corresponding easiest function, we established a lower bound on the (1+1) CHM ’s performance on any function. We also showed that MinBlocks is exponentially hard for the standard (1+1) EA, complementing the known result that the (1+1) CHM performs asymptotically worse by a factor of ${\Theta \left( n\right) }$ compared to the (1+1) EA on OneMax. Furthermore, we proved that several hybrid algorithms combining the (1+1) CHM and the (1+1) EA solve both MinBlocks and OneMax only at a constant factor slower than the pure algorithms.

Experimental work revealed that a fitness function consisting of a weighted combination of MinBlocks and OneMax is easy to optimise for both pure operators and hybrid variants even when the OneMax weight component is very small. Nevertheless, after providing the exact fitness landscape of the easiest function for the (1+1) HA(1 / 2,1 / 2), $\textsc {EasiestHybrid}_{1/2}$ for small instance sizes, we observed that its structure is more complex than a simple weighted combination of OneMax and MinBlocks. We leave constructing and analysing easiest functions for other operators that fit the (1+1) $A$ scheme for future work. Similarly, the question about the easiest functions for different schemes of hybridisation remains open.

## Footnotes

1 In drift analysis, a drift function is a non-negative function *d*(*x*) such that $d(x)=0$ for an optimal point and $d(x)\ge 0$ for a non-optimal point. The expected drift at point *x* is ${\text {E}\left( d(x) - d(y)\right) }$, where *y* is the next iteration’s search point. The expected drift is denoted by $\varDelta _f(x)$ when maximising *f*(*x*), or $\varDelta (x)$ in short.

2 Hardest functions are formally only defined for functions and algorithms with finite expected optimisation times. However, we regard functions leading to infinite expected times as being harder than those with finite expected times.
